# Beyond peripersonal boundaries: insights from crossmodal interactions

**DOI:** 10.1007/s10339-023-01154-0

**Published:** 2023-09-01

**Authors:** Gianluca Finotti, Dario Menicagli, Daniele Migliorati, Marcello Costantini, Francesca Ferri

**Affiliations:** 1 Centre for Studies and Research in Cognitive Neuroscience, Department of Psychology, University of Bologna, Via Rasi e Spinelli 176, 47521 Cesena (FC), Italy; 2 MOMILab, IMT School for Advanced Studies Lucca, Lucca, Italy; 3 Scuola di Specializzazione in Psicoterapia Cognitiva e Comportamentale, Associazione di Psicologia Cognitiva, Rome, Italy; 4 TEAM Lab, University G. d’Annunzio, Chieti, Italy; 5 Institute for Advanced Biomedical Technologies ‐ ITAB, Foundation University G. d’Annunzio, Chieti, Italy

**Keywords:** Peripersonal space, Multisensory integration, Crossmodal congruency, Bodily-self consciousness

## Abstract

We experience our self as a body located in space. However, how information about self-location is integrated into multisensory processes underlying the representation of the peripersonal space (PPS), is still unclear. Prior studies showed that the presence of visual information related to oneself modulates the multisensory processes underlying PPS. Here, we used the crossmodal congruency effect (CCE) to test whether this top-down modulation depends on the spatial location of the body-related visual information. Participants responded to tactile events on their bodies while trying to ignore a visual distractor presented on the mirror reflection of their body (Self) either in the peripersonal space (Near) or in the extrapersonal space (Far). We found larger CCE when visual events were presented on the mirror reflection in the peripersonal space, as compared to the extrapersonal space. These results suggest that top-down modulation of the multisensory bodily self is only possible within the PPS.

## Introduction

Specific mechanisms integrate multisensory information from the space close to the body, the peripersonal space (PPS), with tactile information on the body (Graziano and Cooke 2006). Increasing evidence suggests that multisensory coding of peripersonal space is important for a vast spectrum of functions including body representation, goal-directed movements, defensive movements, and social interactions. These functions possibly rely on the top-down modulation of multisensory integration mechanisms underlying the PPS representation (Heed et al. 2010; Pellencin et al. 2018). Salomon et al. (2012) showed that presenting an individual with the image of a body modulates the integration of visuo-tactile stimuli, and this effect is enhanced with images of the individual’s body. These findings leave open the question as to whether the location of the visual information related to one’s body (within or outside the PPS) determines whether this information will modulate the visuo-tactile integration. In other words, does the image of one’s body presented outside the PPS affect the integration of visuo-tactile information differently from when it is presented within the PPS? If this was the case, it would imply that there is a partial overlap between the processing of visual self-related information and peripersonal/extrapersonal space representation. Moreover, answering this question would increase our knowledge of how the brain performs several daily tasks, such as guiding our actions through a mirror. For instance, even a simple action such as shaving requires a complex integration of visual information with sensory and proprioceptive information originating from the skin surface and our joints and muscles. Seeing our image in the mirror either within or outside the PPS could determine whether the visual information is remapped back to our own body, thus affecting multisensory integration processes.

The present study aims to shed light on this issue by taking advantage of a well-established behavioural effect, i.e., the Crossmodal Congruency Effect (CCE).

In a typical crossmodal congruency task (for a review, see Spence et al. 2008) participants hold two foam blocks, one in either hand, with embedded vibrotactile stimulators and light emitting diodes (LEDs) in the upper and lower surfaces. On each trial, a vibrotactile and a visual stimulus are presented randomly from any one of the two possible stimulus locations (up/down). Participants are required to make speeded elevation judgement for each vibrotactile target stimulus, presented to either the index finger or the thumb, while simultaneously ignoring any visual distractor. The common finding is that participants are significantly faster at judging the elevation of tactile targets when visual distractors are presented at the same elevation (congruent trials) than when distractors occur at opposite elevations (incongruent trials). The performance difference between incongruent and congruent trials is known as the CCE, which is considered a reliable measure of multisensory processing (Spence et al. 2008; Spence and Driver 1998). Most interestingly, CCE can be reliably used as a measure of crossmodal mapping (Spence et al. 2004a, b) and remapping of peripersonal space as a function of online sensory-motor requirements (Brozzoli et al. 2010; Lohmann et al. 2019). There is plenty of evidence indicating that the CCE is modulated by the distance of the distractors from the body (Maravita et al. 2002a, b, 2003; Pavani and Castiello 2004; Schicke et al. 2009; Spence et al. 2004a, b). Specifically, the CCE is larger when the distractors are located close to the stimulated hands rather than far away (Spence et al. 2004a, b).

Furthermore, CCE is also enhanced when distractors are presented near objects that the brain currently regards as belonging to the body (Graziano 1999; Iriki et al. 1996; Maravita et al. 2002a, b; Pavani et al. 2000) for instance, near a rubber hand—provided that it is spatially aligned with the participant’s hand (Pavani et al. 2000)—or near the image of one’s own back during a whole-body illusion (Aspell et al. 2009), and, Heed et al. (2010) showed that perceiving a partner’s action in one’s PPS reduces visuo-tactile integration.

Interestingly, Salomon et al. (2012), investigated the idea that visuo-tactile integration is modulated by the presentation of visual information related to human bodies and, in particular, by the identity of the body presented. They used a crossmodal congruency task in which the distractors were presented on a large screen placed in front of participants displaying either a photo of the participant or that of another person and found that CCEs were enhanced in the “self” condition. However, the authors did not test whether the effect disappears if the image of the participant’s body is presented outside their PPS.

Overall, these results from studies using CCE suggest a close relationship between multisensory integration, peripersonal space, and bodily representations of self and others.

Here, we present an experiment aimed at investigating the modulation of the CCE induced by visual information related to one’s body or another’s body, in the peripersonal space vs the extrapersonal space. Such investigation might shed new light on the mechanisms underlying self-other distinction in the PPS.

In this study, participants performed a CCE task in which they saw the spatially congruent and incongruent visual distractors on the mirror reflection of their bodies. The mirror reflection was presented either within or outside the participants’ PPS.

Given the strong modulation of the CCE for distractors presented on bodily self-related stimuli (Aspell et al. 2009; Maravita et al. 2002a, b; Pavani et al. 2000; Salomon et al. 2012) we expected to find larger CCE with one’s mirror reflection image within the PPS as compared to outside the PPS.

A possible limitation of this study is that, even if larger CCE were found in the PPS, it does not rule out the possibility that this effect is not specific to seeing oneself in the mirror but is caused by seeing anyone’s image. For this reason, we performed a control experiment to further test whether this effect was specific to seeing self-related stimuli in the mirror or was equally present when seeing someone else’s reflection in the mirror. In this control experiment, participants performed the same task but were presented with the mirror reflection of another person’s body either within or outside the participants’ PPS. We expected that the difference in CCE would be reduced or abolished when the viewed mirror reflection was that of another person’s body within or outside the PPS of the participant (Control Experiment). Importantly, to avoid any order effect, the administration of Experiment 1 and the Control experiment was counterbalanced across participants.

As we said, this control experiment was designed to better understand whether any modulation of the CCE by the distance of the person reflected in the mirror was specific for self-related stimuli. However, as we will explain later, some features of this control experiment (i.e., the visual appearance of the stimuli) make it problematic to directly compare the results from the two experiments. For this reason, we have refrained from doing so and results from experiment two can only indirectly support our claim on the specificity of the results of our main experiment. For more on this, see the Discussion section.

## Experiment 1

### Methods

#### Participants

Thirty-five volunteers (9 males, mean age = 19.5 years, SD = 2.1, range 18–25) participated in the study. The main analysis reported in this manuscript is the repeated measures ANOVA. For this analysis, the effect size of the interaction effect is *η*^2^_p _= 0.11. Based on this effect size, post hoc power analysis using G*Power software (Erdfelder et al. 1996) shows an achieved power (1−$\beta$ error probability) = 0.32. This shows that there was a considerable probability of committing a type II error (false-negative). Given that we observed the expected effect of Congruency both in Experiment 1 and our control experiment, this eventuality did not occur.

Participants were all right-handed and had normal, or corrected-to-normal vision, and normal touch sensibility as self-reported. They took part in the study after providing written informed consent.

#### Apparatus and stimuli

Participants sat in front of a vertical mirror measuring 120 cm in height and 30 cm in width. Tactile stimuli were delivered via magnetic solenoids with a 2 mm wide tip. The solenoids were attached to the participant’s body in four different locations: right elbow, left elbow, right wrist, and left wrist. Visual stimuli were delivered via ultra-bright red light emitting diodes (LEDs, 5 mm diameter, 50 lm/m^2^), placed on the same body parts of the participant’s body. Both tactile and visual stimuli were clearly above the sensory threshold and lasted 30 ms. The timing of the stimulation and participants’ responses were controlled via an Arduino Uno connected to a PC running Psychtoolbox (Brainard and Brainard 1997).

#### Procedure

The participant was asked to wear a blue t-shirt and a white cross was positioned on the participant’s chest, parasagittal to their nose. A mirror was positioned in front of the participant, perpendicularly to the participant’s sagittal plane, 25 cm (*Near* conditions), or 90 cm (*Far* conditions) from the participant’s eyes.

**Fig. 1:**
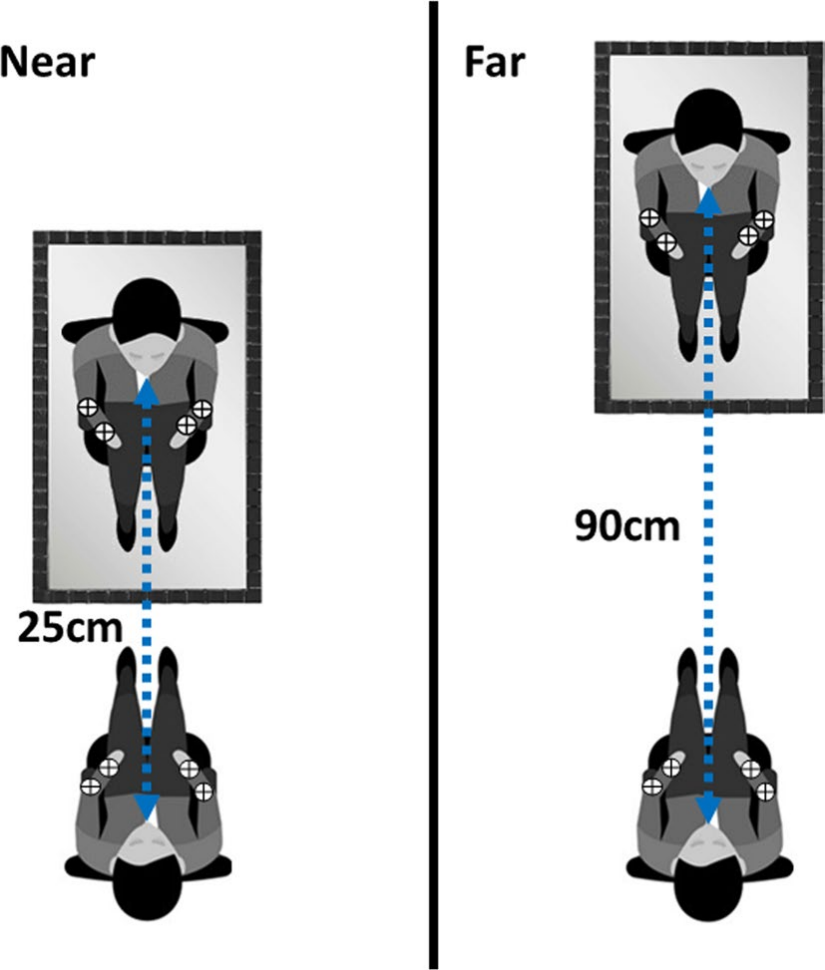
Representation of the experimental design. The rounded blue shapes represent the position of the mirror with respect to the participant. The dotted line with double arrows indicates the distance in centimetres between the participant’s eyes and the mirror. In the near condition, the mirror was located 25 cm from the participants’ eyes, in the far condition it was located 90 cm from the participants’ eyes. These numbers refer to the physical distance of the mirror with respect to the participants. The perceived distance was 50 and 180 cm, respectively, for the near and the far conditions. In both conditions, participants responded to tactile targets while trying to ignore the visual distractors

Given the physical properties of mirrors, the reflected image (referred to as virtual image) is not perceived on the surface of the mirror, instead, it is projected as if the virtual image was originating from behind the mirror. In flat mirrors, the image between an object and the mirror is the same as the distance between the mirror and the virtual image. Hence, the perceived distance of the visual stimuli and fixation cross is calculated by doubling the distance between these stimuli and the mirror. Thus, the visual stimuli appeared to the participants as if placed a total distance of 50 cm in the near condition, and 180 cm in the far condition (Fig. 1).

Participants were asked to focus on the mirror reflection of the fixation cross throughout the experiment and the experimenter monitored the participants via the mirror. This was done to ensure that, overall, they were paying attention to the experimental stimuli and that they were looking at the fixation cross at the beginning of each trial and not to track the eye movements as we did not believe that this was necessary for the current study. Participants performed a modified version of the spatial compatibility effect. They were asked to report, as fast as possible, the side of the tactile target, (left or right), while trying to ignore the visual distractor delivered on their body.

Responses were provided by pressing one of two buttons on a response pad (Cedrus RB-844) with the left or the right index finger, depending on the perceived location of the tactile stimulus. During catch trials, only the visual distractor was delivered, while the tactile target was omitted. Accuracy and reaction times were recorded.

**Fig. 2:**
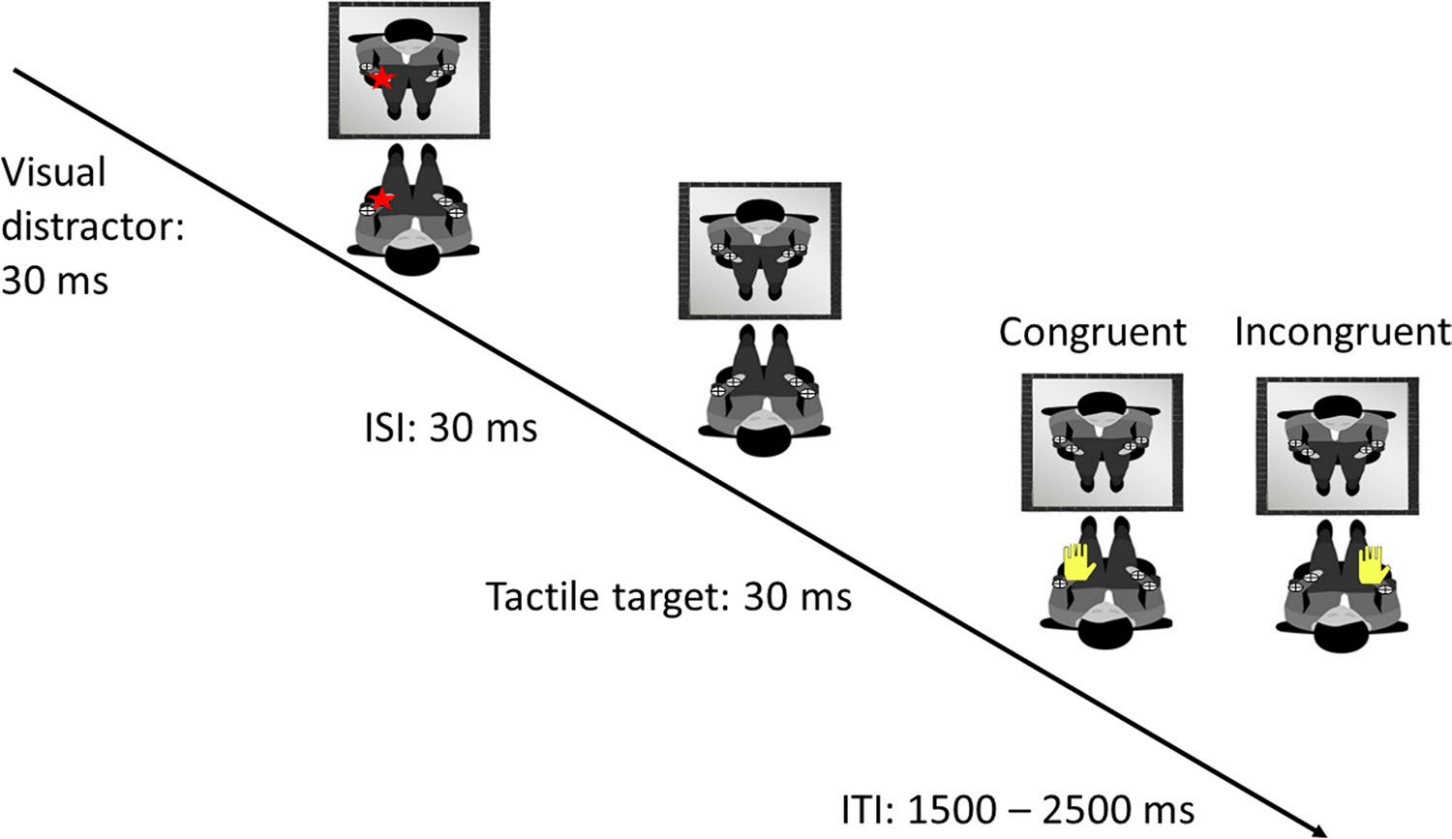
Shows the timeline for a single trial. In this example, first, a visual distactor is presented for 30 ms on the left forearm of the participant, represented with a red star. This is followed by 30 ms Inter Stimulus Interval (ISI), finally, the tactile target was delivered for 30 ms, represented here with a yellow hand. The tactile target was either congruent or incongruent. Given that, in this example, the visual distractor was presented on the left side, the tactile target would have been presented on the left arm in a congruent trial or on the right arm in an incongruent trial. The Inter Trial Interval was variable between 1500 and 2500 ms

Each participant performed two blocks, one for each experimental condition (i.e., Self-Near, Self-Far). Each block was made of 172 trials. Each trial was formed by (1) 30 ms visual distractor presentation; (2) 30 ms inter-stimulus interval; (3) 30 ms tactile target stimulation (see Fig. 2).

The inter-trial interval (ITI) was variable between 1500 and 2500 ms. Trials could be either congruent or incongruent. In congruent trials, both the tactile target and the mirror projection of the visual distractor were delivered in the same spatial location, i.e., in specular space. In incongruent trials, the visual distractor and the tactile target were delivered on opposite spatial location. Each block consisted of 72 congruent trials and 72 incongruent trials. Twenty-eight catch trials were also intermixed with the experimental trials.

Before the experimental phase, each subject performed a training phase to familiarize with the experimental procedure and guarantee that participants could feel the tactile stimuli on the body and see the visual stimuli in the mirror. The training was comprised of 16 trials (one for each combination of stimuli) and 4 catch trials.

#### Data analysis

Prior to the analysis, data were tested against the normal distribution, and if necessary, they were appropriately transformed. For each participant, mean reaction times (RTs) of correct responses were calculated for each experimental condition. Responses slower or faster than 2 SDs, compared to the individual mean, were treated as outliers, and not considered for further analysis.

As a first step, we performed a 2 × 2 Classical and Bayesian repeated-measures ANOVA with Congruency (Congruent vs Incongruent) and Distance (Near vs Far) as within-subject factors. The significant threshold for the Classical ANOVA was set to 0.05. For the Bayesian ANOVA, we report the Bayes Factor for all analyses as the probability associated with the alternative hypothesis over the null hypothesis (BF_10_). The default number of Monte Carlo samples was always 10,000. If the ANOVA revealed a significant interaction of Congruency and Distance, we used Estimation Statistics to quantify the effect. In particular, according to the literature (Maravita et al. 2003; Spence et al. 2000), we calculated the difference in performance between incongruent and congruent trials, hereafter referred to as Crossmodal Congruency Effect (CCE), as a measure of crossmodal interaction, where greater CCE indicates a greater magnitude of crossmodal interaction. We then used estimation statistics to calculate the paired mean difference in CCE values in the Near vs. Far condition. Estimation statistics are based on bootstrapped confidence intervals (CIs) and represented with Cumming estimation plots (Cumming 2014; Ho et al. 2019), which show the raw data for each condition and the paired difference with 95% Bias Corrected accelerated (BCa) confidence interval based on 5000 bootstrap samples. The interpretation was based on the inspection of the estimated difference across conditions and the precision of such estimate (i.e., length of the CI): CIs fully overlapping with 0 were interpreted as indicative of no evidence of effect; CIs not overlapping with 0 were interpreted as indicative of small, moderate, or strong evidence of effect based on the size of the estimated difference and its precision (the longer the CI, the weaker evidence; Calin-Jageman and Cumming 2019; Cumming 2014). Estimation statistics were computed using the web application available at https://www.estimationstats.com/ (Ho et al. 2019). Data analysis were performed using JASP (Love et al. 2019); plots were made in R software (Allen et al. 2019) and RStudio.

#### Results

On average, participants did not respond correctly to 3.4% (SD 4%) of the trials. Erroneous trials were discarded and not considered for further analyses. Participants erroneously responded to 2.1% of the catch trials.

The Shapiro–Wilk’s test (*p* < 0.05) (Shapiro and Wilk 1965) showed that RTs violated the assumption for normality (skewness = 1.7, SE = 0.02; kurtosis = 5.9, SE = 0.03), hence RTs were log-transformed.

**Fig. 3:**
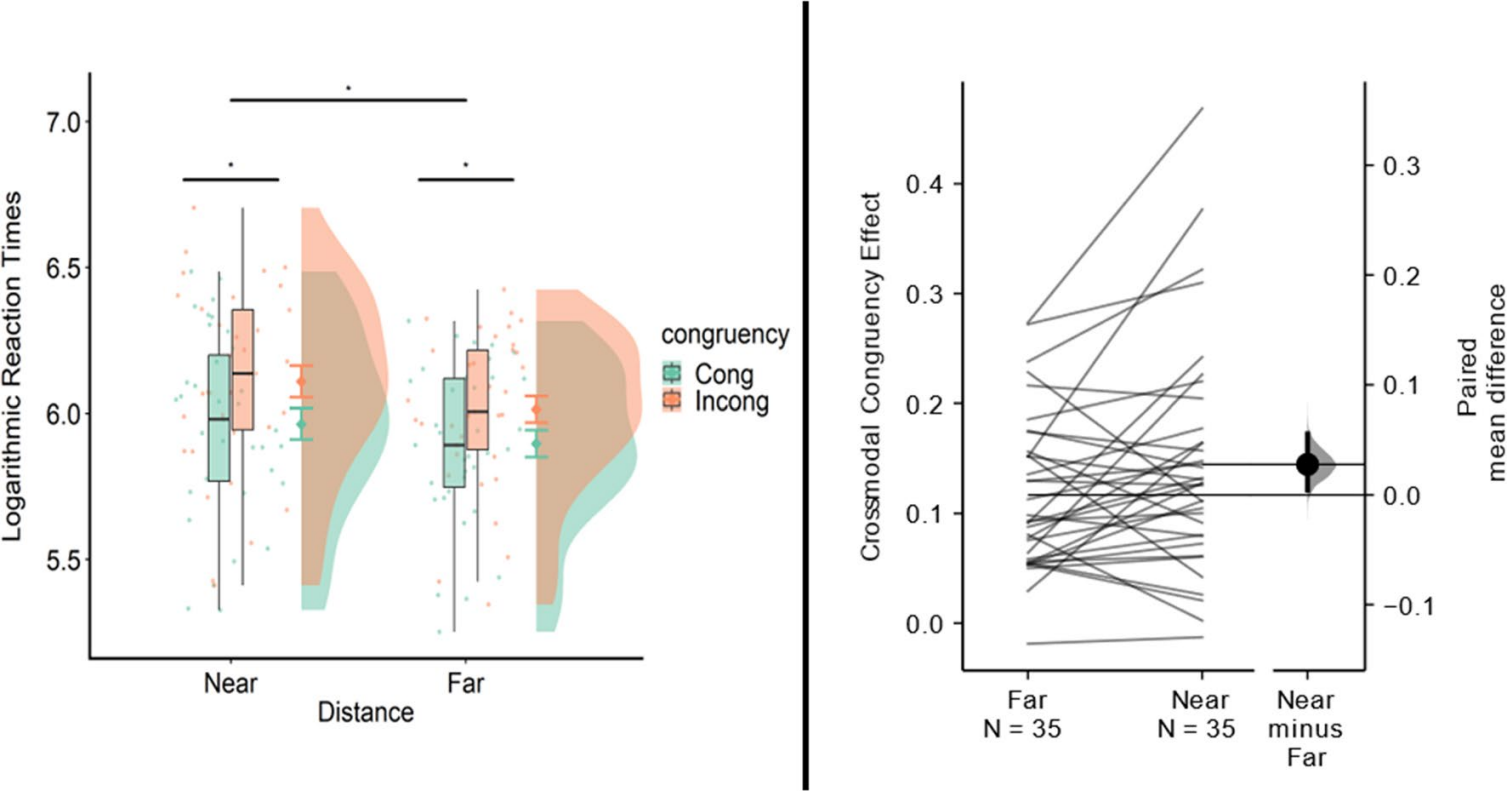
The raincloud plots show data distribution, the central tendency by boxplots and the jittered raw data. Error bars show SEM for each condition. The left panel shows log transformed reaction times across experimental conditions. The horizontal lines with asterisks show the significant main effects. The right panel shows, on the left, the crossmodal Congruency Effect (CCE) values in the Near and Far condition; each paired set of observations is connected by a line. The right side of the plot shows the paired mean difference between CCE scores in the Far and the Near condition plotted as a bootstrap sampling distribution. The mean difference is depicted as a dot; the 95% confidence interval is indicated by the ends of the vertical error bar

The ANOVA revealed a significant main effect of congruency, *F*(1, 34) = 94, *p* < 0.001, *η*_p_^2^ = 0.73, showing that, in both conditions, reaction times were higher for incongruent than for congruent trials (see Fig. 3 left plot and Table1).

**Table 1:** Left side: Mean reaction times in milliseconds and log-transformed mean reaction times across experimental conditions for Experiment 1

	Near		Far		CCE	
	Congruent	Incongruent	Congruent	Incongruent	Near	Far
RT (ms)	408	471	376	422	64	46
SD	123	142	96	102	56	31
Log-RT	5.96	6.11	5.90	6.01	0.14	0.12
SD	0.32	0.32	0.27	0.27	0.10	0.07

Right side: Mean CCE values in milliseconds and log-transformed CCE values across experimental conditions

There was also a significant main effect of distance, *F*(1, 34) = 13.67, *p* < 0.001, *η*_p_^2^ = 0.28, showing that reaction times were significantly higher in the Near than in the Far condition. Importantly, the interaction between Congruency and Distance was also significant, *F*(1, 34) = 4.24, *p* = 0.047, *η*_p_^2^ = 0.11. Overall, these results show that the reaction times were significantly higher for incongruent as compared to congruent trials both in the near and in the far conditions (see Table 1) and that the difference between incongruent and congruent trials was higher in the near as compared to the far condition.

These results were further confirmed by the Bayesian ANOVA. This revealed that the data best supports the model that includes the two main effects of Congruency and Distance and the interaction effect between Congruency and Distance (BF_10_ = 1860_,_ err% = 5893), indicating extreme evidence for H1. The results of the estimation statistics comparing CCE scores in the Near and the Far condition show that there was a small difference (*M*_diff_ = 0.027; 95%CI [0.004 0.056]), with higher CCE scores in the near as compared to the far condition (see Fig. 3, right plot and Table 1).

### Control experiment

#### Participants

Participants from experiment 1 also participated in the control experiment in a separate session.

#### Apparatus and stimuli

The experimental apparatus was the same used for experiment 1.

#### Procedure

**Fig. 4:**
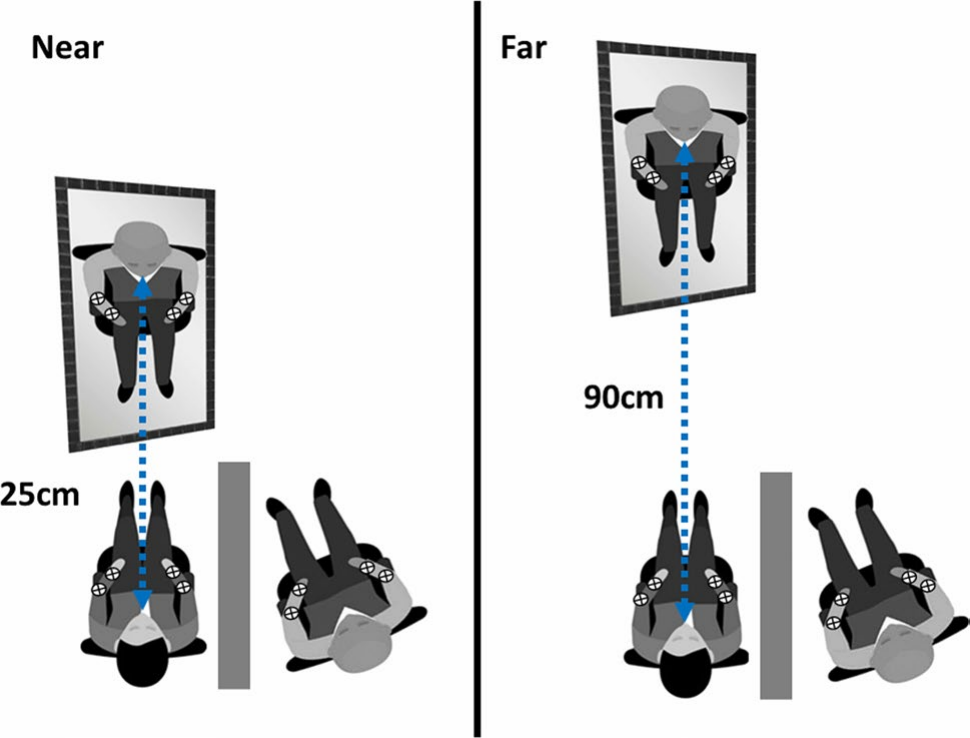
Representation of the experimental design. The rounded blue shapes represent the position of the mirror with respect to the participant. The dotted line with double arrows indicates the distance in centimetres between the participant’s eyes and the mirror. In the near condition, the mirror was located 25 cm from the participants’ eyes, in the far condition it was located 90 cm from the participants’ eyes. In both conditions, participants responded to tactile targets while trying to ignore the visual distractors. The figure also shows the position of the confederate, sitting on the right of the participant, separated by a mobile wall panel

The procedure was the same as in experiment 1 with the only difference that in this session participants were looking at the mirror reflection of a confederate (stranger to the participant). To achieve this, the participant and a confederate were side by side, with a mobile wall panel separating them to prevent direct interaction (see Fig. 4). Both the participant and the confederate were wearing a blue t-shirt and a white cross was positioned on the participant’s chest, parasagittal to their nose. The mirror was rotated by 30 degrees so that it would reflect the confederate’s body. Participants were asked to focus on the mirror reflection of the fixation cross throughout the experiment and were asked to report, as fast as possible, the side of the tactile target (left or right), while trying to ignore the visual distractor. However, differently from experiment 1, the visual distractors were delivered on the confederate body. The number of trials, blocks, and timing of stimulation were the same as in experiment 1.

#### Data analysis

Data were analysed using the same procedure as for experiment 1.

#### Results

On average, participants did not respond correctly to 4.6% (SD 7.9%) of the trials. Erroneous trials were discarded and not considered for further analyses. Participants erroneously responded to 3.0% of the catch trials. As for experiment 1, RTs were log-transformed.

**Fig. 5:**
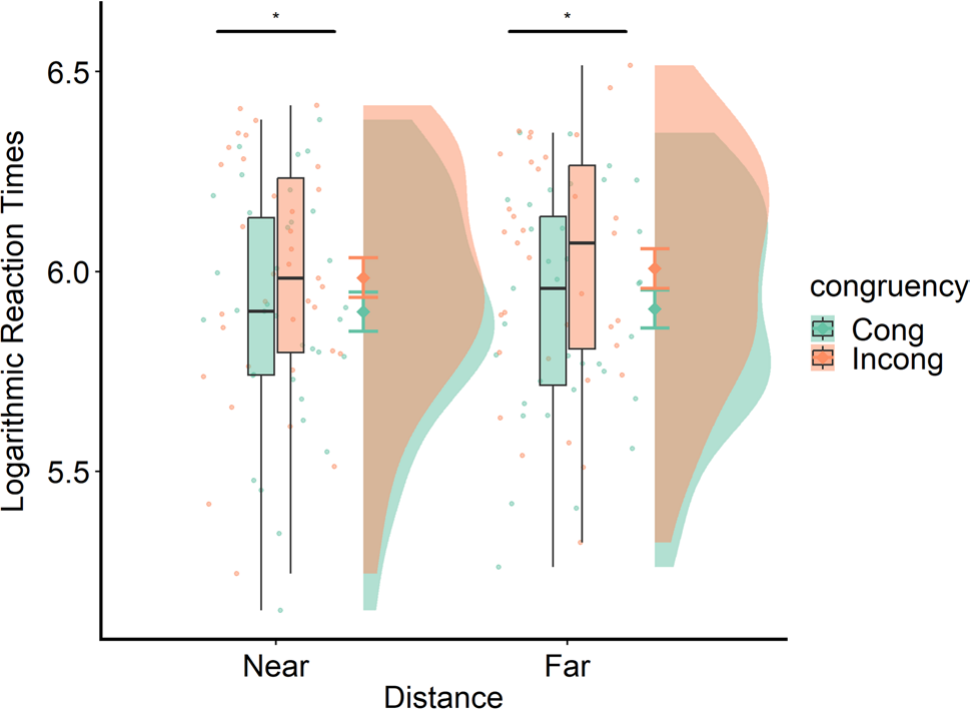
Log transformed reaction times across experimental conditions. The horizontal lines with asterisks show the significant main effects

The ANOVA revealed a significant main effect of congruency, *F*(1, 34) = 48.9, *p* < 0.001, *η*_p_^2^ = 0.59, showing that, in both conditions, reaction times were higher for incongruent than for congruent trials (see Fig. 5, and Table 2). The main effect of Distance was not significant, *F*(1,34) = 0.63, *p* = 0.43, *η*_p_^2^ = 0.01. The interaction between Congruency and Distance was not significant, *F*(1,34) = 3.80, *p* = 0.059, *η*_p_^2^ = 0.1.

**Table 2:** Left side: Mean reaction times in milliseconds and log-transformed mean reaction times across experimental conditions for the control experiment

	Near		Far		CCE	
	Congruent	Incongruent	Congruent	Incongruent	Near	Far
RT (ms)	379	413	381	423	34	42
SD	104	114	102	119	39	38
Log-RT	5.90	5.98	5.91	6.01	0.09	0.1
SD	0.29	0.29	0.28	0.30	0.08	0.08

Right side: Mean CCE values in milliseconds and log-transformed CCE values across experimental conditions

These results were further confirmed by the Bayesian ANOVA. This analysis revealed that the data best supports the model that includes only the main effect of Congruency (BF_10_ = 213,321_,_ err% = 3.12), indicating extreme evidence for H1.

## Discussion

The bodily sense of self can be defined as a sense of self-awareness of one’s own body as a differentiated agent situated in the environment (Rochat 2003). Interestingly such sense of self is not fixed and does not strictly match the boundaries of the physical body, rather it is flexible (e.g., Weser et al. 2017; Finotti et al. 2023) and extends to include what is known as peripersonal space (PPS) (Ferri, Chiarelli et al. 2013; Ferri, Costantini et al. 2013a, b; Pellencin et al. 2018). For instance, the PPS can expand to include an embodied tool, showing a tight link between body representation, PPS and multisensory integration processes (Spence et al. 2004a, b; Grivaz et al. 2017).

Some aspects can enhance or constrain the malleability of the PPS, such as the matching of current visual information with pre-existing knowledge about our own visual identity. When the visual information matches our previous knowledge about our appearance, the crossmodal mapping of peripersonal space is enhanced (Salomon et al. 2012). However, is this crossmodal mapping also enhanced when stimuli are presented outside the peripersonal space? Answering this question would increase our understanding of how the brain processes information during actions that are visually guided via a mirror. Furthermore, understanding this would be important for virtual reality (VR) applications. VR is becoming increasingly popular and available for many applications, such as surgery (Sengül et al. 2012), rehabilitation (Levac and Sveistrup 2014; Schultheis and Rizzo 2001), and even psychotherapy (e.g., Riva 2005; Glantz et al. 2003). This technology has created the opportunity to see oneself (or an avatar resembling one’s body) projected in front of us in the VR space. Knowing how our brain treats one’s visual reflection coming from a mirror in the real world, would provide us with some indication pertaining whether and how an image of ourselves in a virtual environment would be treated, depending on the distance from one’s first-person perspective.

In this paper, we investigated whether visual events on a body, either located in the participant’s PPS or extrapersonal space, interact with the sense of touch on the participant’s body. Results revealed larger CCE when visual events were presented on the mirror reflection near rather than far from the participants. To test for the specificity of the effect, in a control experiment we showed that the CCE is also present when looking at a mirror reflection of someone else, but it is not modulated by the distance of the mirror reflection of the body inside as compared to outside the PPS.

Past research suggests that seeing oneself in a mirror can directly affect perceptual body representations (Preston et al. 2015). Starting from a young age, humans learn to recognize themselves in the mirror and to use it to guide their actions. As Preston and colleagues argued, this understanding suggests that our brain has special mechanisms that go far beyond mirror recognition but also include special transformation processes that translate the visual information back to our own body, thus influencing multisensory integration. That is, according to this view, when looking at oneself in the mirror, the visual information is combined with tactile and proprioceptive information according to an egocentric spatial reference frame centred on one’s body. The data presented in our work suggest that this remapping and integration is higher when the image is presented within the PPS.

It might be argued that larger CCE within the PPS could not specifically reflect the topdown modulation of the multisensory bodily self. To control for this possibility, we performed a control experiment where participants were looking at a mirror reflection of another’s body. Our data revealed a consistent CCE in both experimental conditions, however, in contrast with our main experiment, in the control experiment participants tended to have lower CCE in the near than in the far condition, although this difference was at a trend level and Bayesian analysis indicates that the most relevant factor was Congruency, whereas Distance does not seem to play a role neither by itself nor in interaction with Congruency. The fact the participants in the control experiment tended to show a different pattern of results might support the idea that the identity of the reflection in the mirror plays a role in modulating the CCE with enhanced multisensory processing for bodily-self representation. However, it should be noted that we did not and could not directly compare results from the two experiments, as bottom-up visual features of the stimulation inevitably do not correspond. This is a limitation of our study that should be further addressed in future experiments. A possible solution to this problem could be to use mixed reality to present the stimuli. Mixed reality allows the incorporation of virtual graphics into a real three-dimensional scene or, conversely, the inclusion of real-world elements into a virtual environment. Using this technology, it would be possible to project in front of the participants either an image of themselves or that of another person without changing the angle of the image. In other words, this would allow the experimenter to selectively manipulate the identity of the person that the participant is seeing without changing the low-level features of the stimuli.

Our findings seem to be consistent with evidence showing that stimuli delivered on one’s body receive preferential treatment by the brain, producing faster and more accurate behavioural responses. The visual remapping of touch is a clear example of these effects (Cardini et al. 2011; Serino et al. 2008). In the visual remapping of touch, perception of near-threshold tactile stimuli on the face is modulated if participants observe a face being touched. This effect is specific to viewing a bodily stimulus because the effect of vision on touch disappears if participants observe the picture of an inanimate object (Cardini et al. 2011; Serino et al. 2008). Importantly, the effect of vision on touch is maximum when subjects observe their face being touched, instead of the face of another person (Cardini et al. 2011; Serino et al. 2008).

Similarly, seeing a hand improves tactile acuity, even when such vision is entirely non-informative regarding the tactile stimulus (Beck et al. 2015; Longo et al. 2008, 2011). This effect, known as Visual Enhancement of Touch (VET) suggests a relationship between tactile perception and visual perception of body parts. Importantly, research has shown that the VET effect is not driven by the visual stimulus of a hand per se but relates specifically to the representation of the hand as part of the body. For instance, Longo and colleagues (Longo et al. 2008) examined whether VET is modulated by the feeling of owning the observed hand, using the rubber hand illusion. Results revealed larger VET when participants performed the tactile detection task while observing an embodied rubber hand, rather than a non-embodied rubber hand.

Interestingly, perceptual processing is enhanced not only when the stimuli are delivered on or near the one’s body, but also when they are self-related or self-associated compared to when they are not related to one’s self (e.g., Singh and Karnick 2022). Several findings have demonstrated that stimuli that are self-related, such as someone’s own face or name, receive preferential processing across all senses (Moray 1959; for a review see Cunningham et al. 2017), a phenomenon usually referred to as self prioritization effect (SPE). Even making arbitrary stimuli self-related, instantly confers them preferential processing advantages, thus enhancing accuracy and reaction times, proportionally to the social proximity to the self (Scheller and Sui 2022). Not only self-relevance enhances unisensory processing but also multisensory integration. Scheller and Sui (2022) assessed the propensity to experience the sound-induced flash illusion before and after event-information was associated with social identities and found that self-relevance largely modulates the fusion illusion, suggesting that newly learned associations which increase self-relevance modulate multisensory integration. This is in line with our study in which multisensory stimuli are more likely processed together (hence the higher CCE) when in connection with self-related information presented in the near as compared to far space and suggests an interplay between multisensory integration processes, SPE and the peripersonal space which should be further investigated in future experiments.

Our effects are also unlikely to be explained by a saliency effect for the near stimuli, as compared to far stimuli. In fact, the CCE was observed for all experimental conditions in both the main and the control experiments. Moreover, a larger CCE has been observed in experiment 1 for the Self Near condition, but the control experiment showed a trending opposite pattern, that is, a larger CCE for the Far than the Near condition.

Another possibility is that the presence of the experimenter in the room may have affected the results. For instance, using an audio-visual multisensory task, Barutchu and Spence (2020) showed that multisensory enhancements were modulated by the presence or absence of the experimenter in the room, even though the experimenter was not visible to the participants. When participants were left alone in the room, they were less likely to show multisensory enhancements for repeating and familiar congruent stimuli. The presence of an experimenter in the room significantly enhanced multisensory processing for repeating stimuli. The authors speculate that the presence of the experimenter in the room increased participants’ attention, motivation and obedience to task instructions which, in turn, up-regulated multisensory integration to all relevant target stimuli irrespective of stimulus congruence.

We cannot rule out that, also in our experiment, the presence of the experimenter in the room may have had a similar effect. However, it should be noted that in our experiment the experimenter was present in all phases of the testing procedure, hence, any enhancing effect of attention should have been equally observed in all experimental conditions and would not account for the differences observed between the near and the far conditions.

Related to this topic, one could ask whether the presence of a confederate in the room affected the participant’s performance in our control experiment. For instance, previous works have found that in performing a Simon or a Flanker task together with another participant, performance is affected by the presence of the co-actor (Atmaca et al. 2011), even when the co-actor was not visible but the participant knew where they were seated (Dittrich et al. 2017). Even more interestingly for the present study, Hobeika et al. (2020) showed that the PPS can expand during collaborative tasks.

A key difference is that in these experiments both participants were performing the task together, whereas in our work the confederate was merely a presence irrelevant to the task. For these social effects to occur, a crucial factor is whether the co-actor is collaborating with the participant. For instance, Atmaca et al. (2011) showed that the social effect of performing the tasks with another person is only observed when participants perceive the co-actor as intentionally controlling their actions or, in other words, actively participating in the task. Similarly, the social effect on the PPS is not observed when the other participant was merely present or when participants were in competition with each other (Atmaca et al. 2011).

Some important limitations should be considered when evaluating the results of this experiment: first, our sample size was unbalanced for gender, with 26 Females and 9 Male participants, which could potentially limit the generalizability of our results. Another important limitation is that the gender of the confederate was always male, regardless of the gender of the participants which might affect our results in the control experiment. Unfortunately, given that the sample size was unbalanced with only 8 male participants, this does not make it possible to statistically test whether the participant’s gender played a role. However, visual inspection of data does not support this idea: females and males group means follow the same pattern for all conditions both for our main experiment and for the control experiment.

Another aspect of our results that should be considered is that in experiment 1 participants responded more rapidly when the stimuli were in the Far as compared to the Near condition. This is not a limitation per se, but it seems counterintuitive as one would expect participants to respond faster to stimuli presented close to their bodies. Unfortunately, with the current data, it is not possible for us to explain why this is the case and future studies are needed to explain this.

What our data add to the current literature is that the effect of visual perception of own body on tactile perception is stronger when the self-related image is observed within the peripersonal space. Self-images projected outside the peripersonal space produce a smaller behavioural effect. The definition of PPS (Rizzolatti et al. 1981) originates from electrophysiological studies based on visual–tactile neurons identified in the premotor and the ventral intraparietal area (VIP) of the monkey brain (Fogassi et al. 1999; Graziano 2001; Rizzolatti et al. 1997). The receptive fields of the neurons of this fronto-parietal network are coded in somatic coordinates and anchored to various parts of the body. In particular, the visual receptive fields of premotor neurons around the hand extend from 5 to 35 cm from the tactile receptive fields (Fogassi et al. 1999). The advantage of this mapping of space, which seems also to be a characteristic of humans (Brozzoli et al. 2011; Chieffi et al. 1992; Makin et al. 2007), is quite obvious: it enables efficient mapping of what is near, thus permitting us either to take advantage of an opportunity or to avoid a threat.

There are circumstances in which the distinction between the self and the other is blurred. This is, for instance, a common self-disorder symptom in schizophrenia. To date, self-disorder symptoms are explicitly assessed by using questionnaires. The new task proposed in this paper could be used to implicitly examine the self-other distinction in self-disorders. Previous research found that Schizophrenic patients show reduced intersensory facilitation (i.e., faster reaction times for cues from two sensory modalities compared to unimodal targets), lower temporal resolution in the integration of multisensory stimuli (e.g., Williams et al. 2010) and smaller peripersonal space boundaries in schizophrenic patients as compared to healthy controls (Di Cosmo et al. 2018; Lee et al. 2021). Finally, it has been found that these patients show earlier self-recognition and delayed other recognition, thus appearing more centred on their own image compared to healthy controls (Keromnes et al. 2018). Taken together, this evidence depicts a complex picture that might suggest that, if tested with the paradigm presented in the present work, schizophrenic participants would show reduced CCE scores and a higher role of the distance of the self-image compared to healthy controls. Given the complex interaction between these factors, future experiments should test how schizophrenic patients would perform when tested with this paradigm.

## Data Availability

All the data collected for this experiment are available online on Open Science Framework at: https://osf.io/2da54/files/. Beyond Peripersonal Boundaries: Insights from Crossmodal Interactions.
